# Effect of Injection Molding Parameters on the Tensile Strength of Short-Carbon-Fiber-Reinforced Nylon 6

**DOI:** 10.3390/polym17091264

**Published:** 2025-05-06

**Authors:** Runtian Zhao, Xiaodong Li, Zhihui Wang, Ting Wu, Jianguo Liang

**Affiliations:** 1 College of Mechanical and Vehicle Engineering, Taiyuan University of Technology, No. 79, Yingze West Street, Taiyuan 030024, China; tyutzrt@163.com (R.Z.); lxd15713535671@163.com (X.L.); 17703408774@163.com (Z.W.); 2 Advanced Forming and Intelligent Equipment Research Institute, Taiyuan University of Technology, No. 79, Yingze West Street, Taiyuan 030024, China; wuting01@tyut.edu.cn

**Keywords:** injection molding, influence mechanism, composites, mechanical properties, nylon 6

## Abstract

SCF/PA6 composites have gained extensive industrial applications due to their superior processability and moldability, with long-fiber pellet injection molding being the predominant manufacturing technique. However, systematic investigations into injection molding parameter optimization and its mechanistic impacts on tensile strength remain scarce. This study employed the Taguchi method to investigate the effects of four critical process parameters—injection pressure, melt temperature, mold temperature, and injection time—on the tensile strength of short-carbon-fiber-reinforced nylon 6 (SCF/PA6), while elucidating their underlying mechanisms. The optimal parameter combination within the experimental range was determined to be an injection pressure of 100 bar, a melt temperature of 280 °C, a mold temperature of 100 °C, and an injection time of 2 s. Under these optimized conditions, the tensile strength reached 184.33 MPa, representing an 8.05% enhancement over baseline values. Mechanistic analysis revealed that melt temperature and injection time (essentially reflecting injection velocity) primarily govern fiber orientation distribution. Notably, melt temperature additionally regulates molecular chain orientation in the amorphous matrix regions. Injection pressure predominantly influences process-induced defect formation and material densification. Mold temperature exhibits a negligible impact on tensile strength.

## 1. Introduction

Carbon fiber (CF) possesses excellent properties such as high strength, high modulus, light weight, good chemical stability, heat resistance, and a low thermal expansion coefficient [1,2,3,4]. In comparison to continuous-fiber-reinforced thermoplastic resin, short carbon fiber (SCF)-reinforced thermoplastic resin (SFRP) offers the advantages of low cost, excellent formability, and easy recovery [5,6,7]. Nonetheless, its low strength stands as the primary factor restricting the development of its application, making the enhancement of strength a top priority for the development of SFRP [8,9].

The primary factors influencing the strength of SFRP are the mechanical properties of raw materials and the molding process. In terms of raw materials, polyamide 6 (PA6) is a high-performance semi-crystalline polymer with good impact resistance, friction resistance, thermal stability, and ease of processing [10]. As a result, it is widely utilized in the automotive industry and is often employed as a matrix material for SFRP [11]. Regarding the molding process, extrusion pelletization and injection molding are the mainstream processes due to their advantages of high efficiency, low cost, and good molding quality [12,13]. The entire process can be divided into two stages. The first stage is the extrusion pelletization stage, where the mechanical properties of the composite material are influenced by altering the screw speed, screw tooth shape, and fiber inlet position of the double worm extruder [14,15]. The second stage is the injection molding stage, where the main process parameters include injection pressure, holding pressure, injection temperature, injection speed, and mold temperature. During this stage, the process parameters primarily affect the mechanical properties of the composites by altering fiber orientation and crystallinity [16,17,18]. The influence mechanism of the parameters of the first-stage process (extrusion pelletization) on the mechanical properties is relatively clear [19,20]. The mechanisms by which injection molding parameters influence mechanical properties are complex. Therefore, further research is required. Currently, the Taguchi method is commonly used for the optimization and analysis of injection molding process parameters. Babur Ozcelik and others [21] employed the Taguchi method to study and optimize the effects of injection parameters such as melt temperature, holding pressure, cooling time, and injection pressure on the mechanical properties of acrylonitrile–butadiene–styrene (ABS) molded products. Emel Kuram et al. [22] used the Taguchi method to study the effects of the number of recycling cycles, melt temperature, mold temperature, injection pressure, and holding pressure on the mechanical properties of glass-fiber-reinforced nylon 6 (PA6-GF). Babur Ozcelik [23] applied the Taguchi method to investigate the effects of melt temperature, holding pressure, and injection pressure on the mechanical properties of polypropylene. Hasan Oktem et al. [24] employed the Taguchi method to study the effects of injection speed, material flow rate, mold temperature, and melt temperature on the warpage and mechanical properties of PC/ABS parts and optimized the parameters. Wen-Chin Chen et al. [25] used the Taguchi method to study the effects of melt temperature, injection speed, holding pressure, holding time, and cooling time on the length and warpage of injection-molded products. Nik Mizamzul Mehat et al. [26] proposed a new constitutive method that addresses multi-response problems using a single response combination through the Taguchi approach, applied to improving the mechanical properties of parts made from recycled PP. Tan Yizong et al. [27] investigated the influence of processing parameters on the tensile properties of injection-molded polystyrene (PS) using the Taguchi method. Shahrul Kamaruddin et al. [28] optimized injection molding parameters using the Taguchi method to enhance the quality characteristics of injection-molded products (plastic trays) made from a plastic blend (75% polypropylene (PP) blend and 25% low-density polyethylene (LDPE) blend). Mirigul Altan [29] determined the optimal injection molding conditions for minimizing the shrinkage rate of polypropylene (PP) using the Taguchi method. Although there has been extensive research on the optimization and influencing mechanisms of injection molding parameters, studies on the tensile properties of SCF/PA6 are still scarce; therefore, related research is needed.

This study involved the preparation of SCF/PA6 by extrusion pelletization and injection molding. The effects of injection pressure, melt temperature, mold temperature, and injection speed on the tensile mechanical properties of the composites were analyzed using the Taguchi method. The crystallinity, tensile fracture, and viscosity of the composites were characterized and analyzed by DSC, SEM, and a rheometer. The mechanism by which each of the above four process parameters influences the mechanical properties of the composites was comprehensively considered and revealed.

## 2. Taguchi Method

The Taguchi method is a practical orthogonal experimental design approach that utilizes orthogonal arrays for test planning [22]. By employing this orthogonal experimental method, the required number of tests can be significantly reduced, thereby improving experimental efficiency and lowering costs. For the injection molding process of composite materials, injection pressure, melt temperature, and mold temperature have been demonstrated as primary factors affecting the mechanical properties of molded products [30,31]. From a rheological perspective, injection speed influences the fiber orientation distribution in short-fiber-reinforced composites, warranting detailed investigation. Therefore, this study selected these four process parameters as variables, each set at three levels, to conduct an L9 (3^4^) orthogonal array experiment as shown in Table 1. Among them, the injection speed is represented by the injection time, and the two are inversely proportional; that is, the smaller the injection time, the greater the injection speed.

The experimental results were subsequently converted into signal-to-noise ratios (SNRs). In Taguchi methodology, the “signal” component represents the desired output characteristic, while “noise” denotes undesirable variations in the output. The SNR quantitatively assesses the deviation of quality characteristics from target values. Three types of SNRs exist: smaller-the-better, nominal-the-best, and larger-the-better. A higher SNR indicates superior signal dominance over noise factors, signifying optimal process parameter levels that yield better quality characteristics. For this investigation, the larger-the-better SNR was employed to maximize mechanical performance. The larger-the-better quality characteristic is computed using the following equation:

(1) $$S/N=-10\mathrm{lg}\left[\frac{1}{n}\left(\sum_{i=1}^{n}\frac{1}{{y_{i}}^{2}}\right)\right]$$

where n is the number of fiber orientation tensor variation data sets, which is equal to 9, and $y_{i}$ is the tensile strength of the *i*th data set.

## 3. Experimental Details

### 3.1. Materials

TZ700s-12K carbon fibers (CFs) (diameter = 7 mm, density = 1.80 g/cm^3^, tensile strength = 4900 MPa, tensile modulus = 230 GPa) were supplied by Weihai Guangwei Composites. B3S nylon 6 (PA6) (density = 1.13 g/cm^3^, tensile strength = 81.64 MPa, tensile modulus = 2.29 GPa) was supplied by BASF SE, with a melting temperature (Tm) of 220 °C.

### 3.2. Extrusion Pelletization and Injection Molding

**Figure 1:**
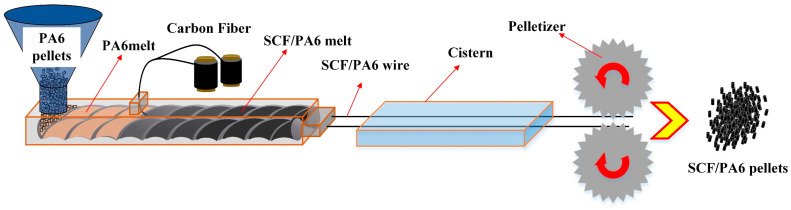
Schematic diagram of the granulation process of long fibers.

The tensile specimens were prepared using an extrusion pelletization and injection molding process, as shown in Figure 1:

Firstly, long-fiber pellets were produced using an SHJ-20 twin-screw extruder (Weidon Machinery Co., Ltd., Nanjing, China) to achieve fiber–resin melt compounding. Due to the high hygroscopicity of PA6, the raw materials were pre-dried at 110 °C in a vacuum oven for 8 h before extrusion. The molten composite was then cooled in a water bath (20 ± 2 °C) for crystallization and subsequently pelletized into granules (3–5 mm in size). During processing, the extruder operated at a screw speed of 85 rpm with a PA6 feed rate of 3.5 Hz, while the barrel temperatures were set to six zones at 235 °C, 245 °C, 250 °C, 250 °C, 250 °C, and 240 °C, respectively, with 4 fiber tows being fed into the system.

**Figure 2:**
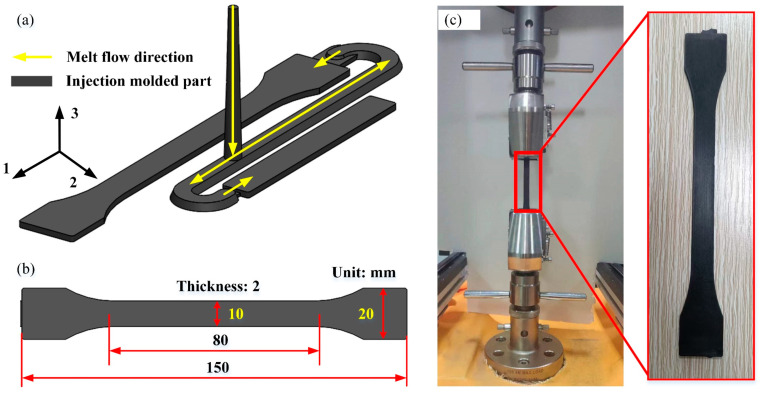
Injection-molded parts and tensile testing process: (**a**) original injection-molded part, (**b**) tensile test samples and dimensions, (**c**) tensile testing process.

The injection molding stage was subsequently conducted using a Changzhou Hongyide HYD-450 vertical injection molding machine, with the molded part shown in Figure 2a. Since this study primarily focused on the tensile properties of the specimens, the sprue, runner, and other excess portions were removed using cutting tools after molding, retaining only the tensile test bars as illustrated in Figure 2b. To ensure molding quality, the composite pellets underwent a secondary drying treatment (110 °C for 8 h) prior to injection to eliminate moisture-induced porosity defects, thereby guaranteeing the reliability of the material’s mechanical performance. The injection molding parameters were configured into nine orthogonal experimental groups according to Table 1.

### 3.3. Characterization

#### 3.3.1. Tensile Strength

Tensile property testing was conducted in accordance with the ASTM D638-14 standard (Test Method for Tensile Properties of Plastics. Springer New York: West Conshohocken, PA, USA, 2014). The tensile experiments were performed using an INSTRON 5969 universal testing machine (ITW Group). The specimens were vertically clamped in the machine’s grips, with one end fixed and the other end stretched at a constant rate of 20 mm/min, as illustrated in Figure 2c. To ensure data reliability, five repeated tests were carried out, and the average value was calculated after the elimination of outliers as the final result.

#### 3.3.2. Crystallinity

The DSC analysis of the samples was carried out in a nitrogen environment using the STA 449 F5 comprehensive thermal analyzer produced by NETZSCH (Netzsch-Gerätebau GmbH, Selb, Germany). The scanning rate was 10 °C/min, and the scanning range was 30–300 °C. The samples were dried at 110 °C for 8 h before the test.

#### 3.3.3. Rheology

The rheometer used in the rheological experiment was the Malvern RH2000 capillary rheometer. The die diameter was 1 mm, the length-to-diameter ratio was 16, the test temperatures were five temperature points: 240 °C, 250 °C, 260 °C, 270 °C, and 280 °C, and the test shear rate range was 20–1000 s^−1^.

#### 3.3.4. Weight Measurement

The tensile specimens were precisely weighed using an FA224TC electronic balance (manufactured by Lichen Company, Changsha, China) with a measurement accuracy of ±0.1 mg.

#### 3.3.5. Microscopic Morphology

The microscopic morphology was characterized by using a Gemini 300 field emission scanning electron microscope (SEM) of Zeiss from Germany. The fiber orientation and fiber distribution of the fracture surface were observed under vacuum conditions. Before imaging analysis, the fracture surface needed to be treated with gold spraying (platinum element), and then the sample was fixed to the base with double-sided tape for observation.

## 4. Results and Discussion

**Figure 3:**
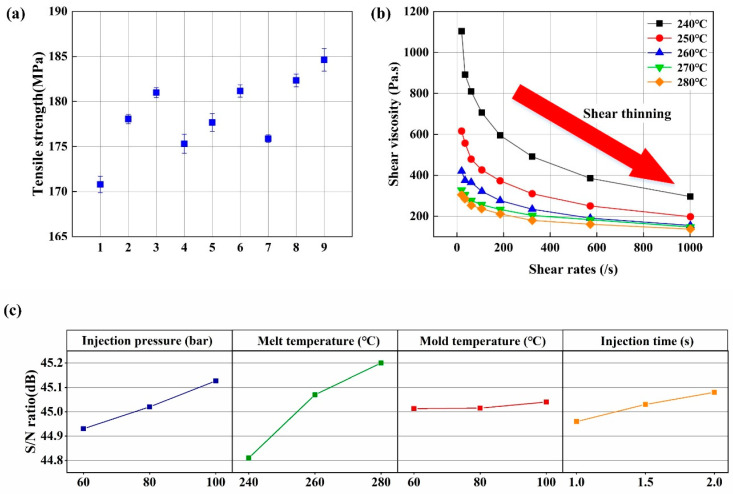
(**a**) Tensile strength of composites, (**b**) melt viscosity–shear rate curves of composites at different temperatures, (**c**) the SNR of tensile strength of composites with different process parameters.

The tensile strength of composites under orthogonal experimental conditions is shown in Figure 3a. As observed, the composite prepared with the first set of orthogonal parameters exhibited the lowest tensile strength of 170.59 MPa, while the ninth set yielded the highest strength of 184.33 MPa—representing an 8.05% improvement. Consequently, the optimal process parameters identified in this study are as follows: an injection pressure of 100 bar, a melt temperature of 280 °C, a mold temperature of 100 °C, and an injection time of 2 s.

The tensile strength variation across orthogonal experimental groups follows a non-linear pattern, as clearly demonstrated in the results. To quantitatively assess the influence of different process parameters on the composite’s tensile strength, SNRs were calculated for each parameter, as presented in Figure 3c. The analysis reveals that the mechanical properties exhibit greater sensitivity to parameters showing wider SNR ranges across different levels. Key observations from the SNR analysis include the following: melt temperature exerts the most significant impact on mechanical performance, injection pressure demonstrates a moderate influence, injection time shows a minimal effect, and mold temperature appears virtually negligible.

Using range analysis, the statistically significant parameters affecting the tensile strength of SCF/PA6 were determined, and the contribution rankings and optimal levels of each control factor in the injection molding process were identified. The range analysis is shown in Table 2. It can be observed that the melt temperature has the largest range and the highest contribution ranking, and the optimal level is 3: 280 °C. Injection pressure ranks second in contribution, with the optimal level at 3: 100 bar. Injection time ranks third, with the optimal level at 3: 2.0 s. The contribution of mold temperature is relatively small and can basically be ignored. This analysis result is consistent with the SNR analysis result.

The above phenomena have been analyzed. The main factors that affect the mechanical properties of the composites are the orientation distribution of the fibers, the density of the product, and the crystallization behavior of the matrix [16,17,18].

### 4.1. Effect of Fiber Orientation Distribution

**Figure 4:**
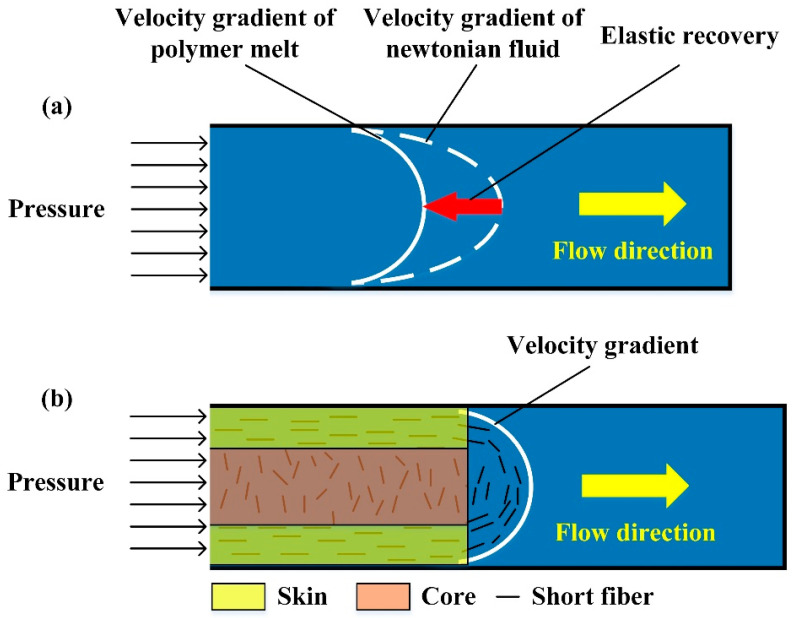
The flow of the composite melt in a pipeline: (**a**) elastic recovery of the polymer melt, (**b**) orientation behavior of short fibers in the melt.

The composite melt is a polymer melt and has obvious viscoelastic properties. Unlike what occurs in a Newtonian liquid, the normal stress *σ*_11_ is generated in the flow direction under shear action, which is equivalent to the melt being stretched in the flow direction [32]. In polymer rheology, the normal stress *σ*_11_ is often calculated by the first normal stress difference *N*_1_, which is expressed as follows:

(2) $$N_{1}={\;\sigma }_{11}\;-{\;\sigma }_{22}=4\eta \dot{\varepsilon }$$

where *σ*_11_ is the normal stress in the flow direction, *σ*_22_ is the stress perpendicular to the flow direction, *η* is the viscosity of the melt, and $\dot{\;\varepsilon \;}$ is the velocity gradient in the flow direction. At a fixed injection speed, the melt in the mold is subjected to the friction of the inner wall to form a determined velocity gradient. According to Equation (2), the lower the melt viscosity of the composite material, the smaller the normal stress in the flow direction of the melt, resulting in a smaller effect of the tensile elongation of the polymer. When the polymer melt is subjected to a greater tensile elongation effect during the shearing process, due to its elastic properties, the relaxation behavior of the polymer molecular chain will make the elastic recovery of the melt in the mold more apparent [33]. Elastic recovery, as shown in Figure 4a, refers to the phenomenon where the flow rate along the diameter of the tube is a flat parabola when the polymer melt flows steadily in the tube. Although the injection mold is more complicated than a circular pipe, the basic law is the same for the tensile specimen mold. The orientation of the fiber in the melt is mainly caused by the shear effect formed by the melt flow rate gradient, as shown in Figure 4b, so the injection-molded parts generally have a skin–core structure. When the parabola of the melt velocity gradient is gentler, the shear effect of the fiber is smaller, and the core will become wider, eventually leading to a decrease in the mechanical properties of the composite.

According to Equation (1), the factors affecting the fiber orientation distribution are the melt viscosity and the melt flow rate gradient during the injection molding process. The melt viscosity of the composite material was measured. As shown in Figure 3b, melt viscosity is affected by melt temperature and shear rate. The melt viscosity showed a nonlinear decreasing trend with the increase in melt temperature, and the gradient of viscosity decrease with the increase in melt temperature was smaller. The effect of melt viscosity on shear rate is also the same. Therefore, an increase in melt temperature will increase the mechanical properties of the composites, and the increasing trend is nonlinear. The mechanism analysis is consistent with the SNR of the melt temperature to the tensile strength of the composites in Figure 3c. The injection time also affects the viscosity of the melt and its flow rate gradient. When the injection time increases, the shear rate of the melt decreases, and the viscosity decreases. At the same time, the melt flow rate gradient decreases. According to Equation (1), the elastic recovery of the melt is proportional to the melt viscosity and its flow rate gradient. Therefore, the mechanical properties of the composites are improved with an increase in injection time. Due to the nonlinearity of the melt viscosity–shear rate curve and the linear effect of the injection time on the melt flow rate gradient, the SNR of the injection time to the tensile strength of the composite also exhibits a small nonlinearity, as shown in Figure 3c.

### 4.2. Effect of Composite Density

When the density of a composite material is higher, it generally indicates that the internal defects—such as air bubbles, voids, or regions of incomplete filling—are minimized. These defects typically weaken the structural integrity of the composite and degrade its overall performance. Among the various processing parameters, injection pressure plays a crucial role in determining the density of the composite material during injection molding. As the injection pressure increases, the molten material is subjected to greater compressive force, which helps to pack the polymer chains more tightly together and reduces the presence of trapped air bubbles. This compression pushes out or eliminates bubbles and voids, resulting in a denser, more homogeneous composite structure.

**Figure 5:**
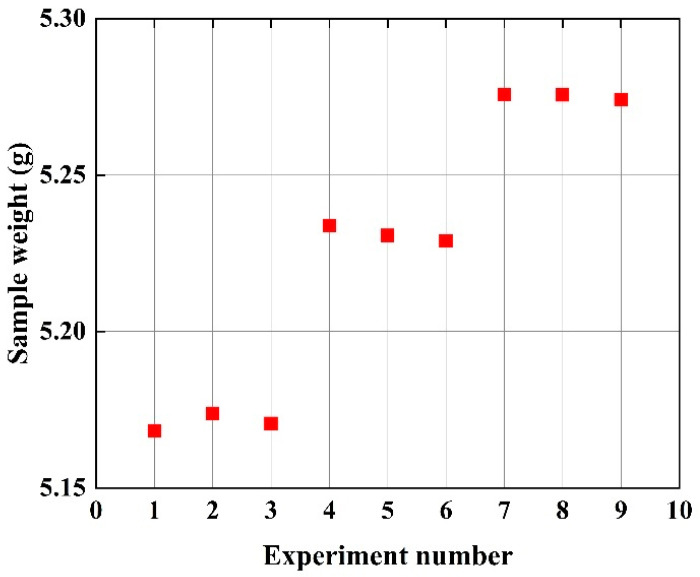
The weight of the sample with different experimental numbers.

This relationship between injection pressure and composite density is experimentally validated by measuring the weight of samples produced under different process parameters. Since the volume of each injection-molded sample remains constant during these experiments, changes in sample mass directly translate to changes in density. Figure 5 illustrates this effect, showing that as the injection pressure increases linearly, the density of the resulting composites also increases approximately linearly. Consequently, this increase in density positively influences the mechanical properties of the composite material, such as its tensile strength. This is further confirmed by observing that the signal-to-noise ratio (SNR) associated with injection pressure and tensile strength similarly shows a roughly linear upward trend, identifying injection pressure as a key factor in enhancing the composite’s performance through improved densification.

### 4.3. Effect of Crystallinity of Composite Materials

**Figure 6:**
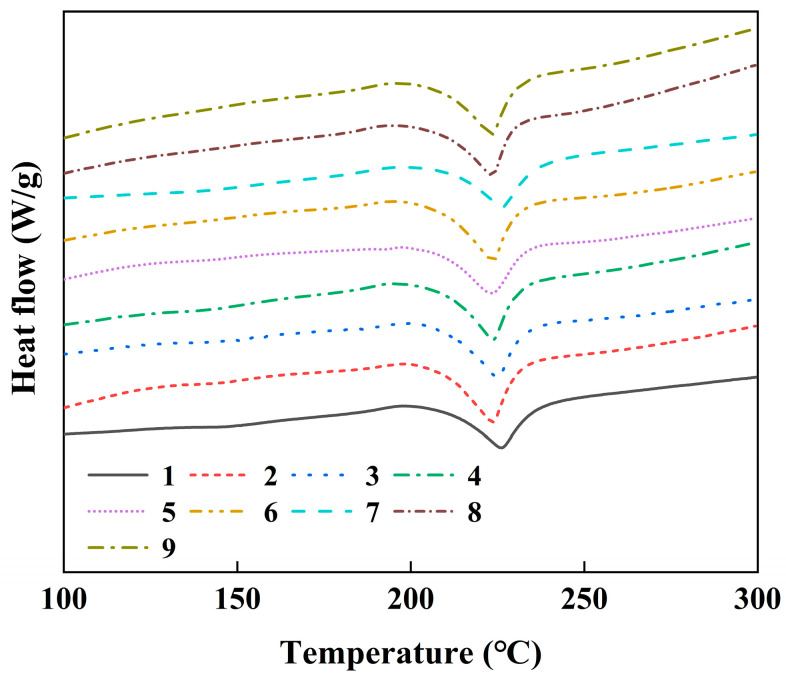
DSC curves of samples with different experimental numbers.

The strength of the polymer increases significantly with the increase in crystallinity, while the toughness decreases [34]. The degree of crystallinity depends on the thermal history and the stress of the polymer during processing [35]. Numerous studies have demonstrated that processing parameters including mold temperature, supercooling temperature (the difference between the melt temperature and the mold temperature), shear rate, and injection pressure significantly influence the crystallinity of polymers [36,37,38]. Therefore, in this study, all parameters will affect the crystallinity of the composite. The composites were analyzed by DSC, as shown in Figure 6, and the crystallinity (χ) of each experimental sample was calculated according to the following equation:

(3) $$\chi =\frac{∆h_{m}}{∆h_{f}^{100}}$$

where $∆h_{f}^{100}$ represents the specific enthalpy of melting of the assumed 100% crystalline material. For polyamide 6, the value is 240 J/g [39]. Detailed results are presented in Table 1, which clearly indicates that the crystallinity levels of the samples produced under various experimental conditions are quite similar across the board. Although slight differences in crystallinity were observed, their small magnitude suggests that crystallinity alone cannot account for the observed differences in tensile strength. Other mechanisms such as fiber orientation and microstructural defects likely dominate. The slight differences in crystallinity can be attributed to the combined influence of the four processing parameters investigated. Each of these parameters interacts in a way that balances the overall effect on the crystallization behavior of the composite material. The underlying mechanisms by which these process parameters affect the crystallinity—including their impact on molecular mobility, cooling rates, and nucleation—have been extensively studied and documented in the existing literature. Given their well-established nature, this study does not revisit these fundamental mechanisms in detail. As a consequence of the relatively stable crystallinity observed in all samples, the signal-to-noise ratio (SNR) related to the mold temperature’s impact on the composite’s tensile strength remains largely unchanged. This suggests that variations in mold temperature within the tested range do not significantly influence the tensile strength through changes in crystallinity, reinforcing the idea that crystallinity is being maintained at a consistent level regardless of adjustments to mold temperature. Thus, mold temperature appears to have a limited direct effect on tensile strength when considered from the perspective of crystallinity alone.

**Figure 7:**
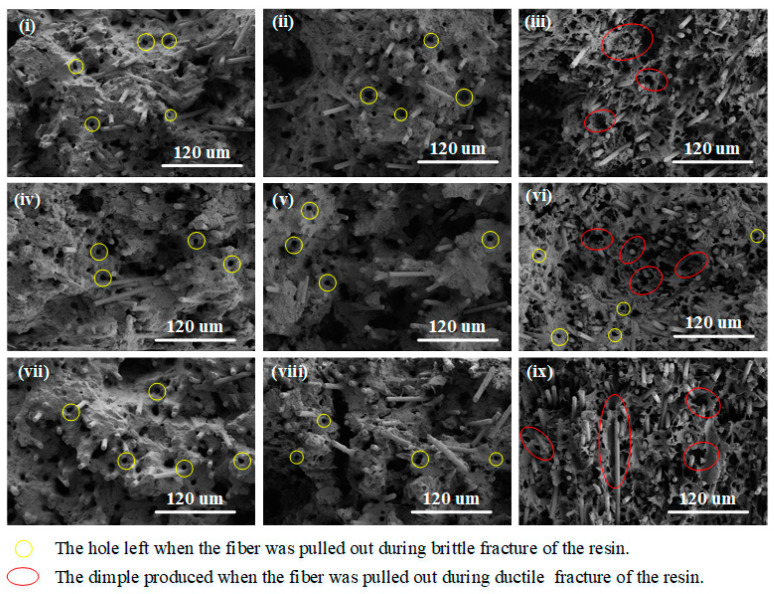
Microstructure of tensile fracture of composites: (**i**–**ix**) correspond to experiment numbers 1–9, respectively.

Combined with the observations presented in Figure 7, it is evident that the matrix in the samples from groups 3, 6, and 9 exhibits clear characteristics of ductile fracture. Specifically, these groups show the formation of dimples around the voids left behind after fibers are pulled out during tensile testing. These dimples are typical signs of ductile failure, indicating that the material underwent significant plastic deformation before fracture. In contrast, the matrix from the other experimental groups shows features characteristic of brittle fracture, where the material breaks suddenly with little to no plastic deformation. This difference in fracture behavior can be attributed primarily to the effect of melt temperature during the injection molding process on the molecular structure of the composite material. When the melt temperature is relatively high, the polymer chains within the amorphous regions of the composite become more mobile and undergo shear forces as the melt flows through the mold. This shear induces the molecular chains to orient themselves along the direction of melt flow. Since, in this study, the melt flow direction coincides with the tensile testing direction, the oriented polymer chains are aligned with the applied load. On the other hand, at lower melt temperatures, the polymer chains are less mobile and remain relatively stable. In this state, the molecular chains tend to be entangled rather than oriented, lacking a preferred direction. This entanglement restricts chain movement and prevents orientation along the flow direction. The implications of this structural difference are significant for the fracture mechanisms observed. When the polymer chains are oriented due to high melt temperatures, the damage mechanism during tensile loading mainly involves the final rupture of these aligned polymer chains, leading to ductile fracture features such as dimple formation. Conversely, when the polymer chains remain entangled and unoriented at low melt temperatures, the matrix damage primarily occurs through displacement and slippage between entangled chains. This displacement accumulates stress and ultimately causes the polymer chains to be cut, resulting in brittle fracture behavior of the matrix.

In summary, the melt temperature during injection molding fundamentally influences the molecular orientation of the composite matrix, which in turn governs the fracture mode exhibited under tensile stress—either ductile fracture with significant plastic deformation or brittle fracture with sudden failure.

## 5. Conclusions

The effect of injection molding parameters on the tensile strength of composites was analyzed by the Taguchi method. The optimal process parameters within the experimental range were obtained using the Taguchi method: an injection pressure of 100 bar, a melt temperature of 280 °C, a mold temperature of 100 °C, and an injection time of 1 s. When the process parameters were changed from an injection pressure of 60 bar, a melt temperature of 240 °C, a mold temperature of 60 °C and an injection time of 1 s to the optimal process parameters, the tensile strength of the composite material increased from 170.59 MPa to 184.33 MPa, with an increase of 8.05%. The influence of injection molding parameters on the tensile strength of the composite was analyzed, revealing the underlying mechanisms by which each process parameter affects the tensile strength. Among these parameters, melt temperature has the greatest effect. As the melt temperature increases, the melt viscosity decreases, which weakens the elastic recovery effect during the injection molding process and ultimately reduces the core size of the injection-molded sample. At the same time, it enhances the orientation of polymer chains in the amorphous region of the matrix, thereby increasing the tensile strength. Injection pressure has the second largest effect. When the injection pressure increases, defects such as bubbles within the composite are reduced, and the resulting increase in density leads to higher strength. The influence of injection time ranks third, which essentially reflects the effect of injection speed. As the injection speed increases, the melt viscosity decreases, and the melt flow velocity gradient increases. This weakens the elastic recovery of the melt, reduces the core size, and improves the material strength. Lastly, mold temperature primarily affects the crystallinity of the composite material. However, since crystallinity is influenced by multiple parameters, its signal-to-noise ratio variation in the Taguchi analysis is comparatively small. The molding process of short-carbon-fiber-reinforced nylon 6 (SCF/PA6) composites can be guided for optimization based on mechanisms by which the obtained process parameters influence tensile strength, enabling further enhancement of material strength. It is believed that this study will provide a useful reference for the injection molding of SCF/PA6 products and promote the practical engineering application of this material.

## Figures and Tables

**Table 1:** The experimentally measured values of the composites on the basis of an L9 orthogonal array in the Taguchi method.

Experiment Number	Injection Pressure (bar)	Melt Temperature (°C)	Mold Temperature (°C)	Injection Time (s)	Tensile Strength (MPa)	Weight (g)	Crystallinity (%)
1	60	240	60	1.0	170.59	5.1683	13.65
2	60	260	100	1.5	178.05	5.1738	14.09
3	60	280	80	2.0	180.98	5.1706	13.70
4	80	240	100	2.0	175.30	5.2338	14.30
5	80	260	80	1.0	177.67	5.2307	13.86
6	80	280	60	1.5	181.17	5.2289	14.67
7	100	240	80	1.5	175.87	5.2757	13.93
8	100	260	60	2.0	182.47	5.2756	13.51
9	100	280	100	1.0	184.33	5.2741	13.53

**Table 2:** Range analysis results.

Factors	Level	Injection Pressure	Melt Temperature	Mold Temperature	Injection Time
K	1	529.83	521.97	535.08	531.75
	2	534.78	538.2	534.51	535.71
	3	541.65	546.06	537.54	538.77
K_avg_	1	176.61	173.99	178.36	177.25
	2	178.26	179.40	178.17	178.57
	3	180.55	182.02	179.18	179.59
Optimal level	-	3	3	3	3
R	-	3.94	8.03	1.01	2.34

## Data Availability

The research data are available upon request to the corresponding author.
